# A quantitative analysis of fidgeting in ADHD and its relation to performance and sustained attention on a cognitive task

**DOI:** 10.3389/fpsyt.2024.1394096

**Published:** 2024-07-01

**Authors:** Ha Min Son, Catrina Andaya Calub, Boyang Fan, J. Faye Dixon, Shahbaz Rezaei, Jared Borden, Julie B. Schweitzer, Xin Liu

**Affiliations:** ^1^ Department of Computer Science, University of California, Davis, Davis, CA, United States; ^2^ Department of Psychiatry and Behavioral Sciences, University of California, Davis, Sacramento, CA, United States; ^3^ MIND Institute, University of California, Davis, Sacramento, CA, United States

**Keywords:** adult ADHD, fidgeting, accelerometer, actigraphy, flanker

## Abstract

**Introduction:**

Attention-Deficit/Hyperactivity Disorder (ADHD) is a neurodevelopmental disorder where hyperactivity often manifests as fidgeting, a non-goal-directed motoric action. Many studies demonstrate fidgeting varies under different conditions as a self-regulating mechanism for attention and alertness during cognitively demanding tasks. Fidgeting has also been associated with reaction time variability. However, a lack of standard variables to define and quantify fidgeting can lead to discrepancies in data and interpretability issues across studies. Furthermore, little is known about fidgeting in adults with ADHD compared to youth. This study aims to design a framework to quantify meaningful fidgeting variables and to apply them to test the relation between fidgeting and performance on a cognitive task, the Flanker, in adults with ADHD.

**Method:**

Our study included 70 adult participants diagnosed with ADHD, aged 18–50 years (30.5 ± 7.2 years). Screening included a structured clinical interview, childhood, current self and current observer ratings of ADHD symptoms. Actigraphy devices were attached to the left wrist and right ankle during completion of a cognitive control, attention task (the Flanker). Laboratory testing was subsequently completed on a single day. The relation between task performance, reaction time variability and fidgeting was examined.

**Results and Discussion:**

Our analysis revealed increased fidgeting during correct trials as defined by our new variables, consistent with previous observations. Furthermore, differences in fidgeting were observed between early and later trials while the percentage of correct trials were not significantly different. This suggests a relation between the role of fidgeting and sustaining attention. Participants with low reaction time variability, that is, those with more consistent reaction times, fidgeted more during later trials. This observation supports the theory that fidgeting aids arousal and improves sustained attention. Finally, a correlation analysis using ADHD-symptom rating scales validated the relevance of the fidget variables in relation to ADHD symptom severity. These findings suggest fidgeting may be a compensatory mechanism that aids in sustained attention for those with ADHD, although alternative explanations exist.

**Conclusion:**

Our study suggests that fidgeting may aid in sustained attention during the attention-demanding, cognitive control processes for adults with ADHD, with more fidgeting observed during correct trials and among participants with lower reaction time variability. Furthermore, the newly defined fidget variables were validated through a significant correlation with ADHD rating scales. By sharing our implementation of fidget variables, we hope to standardize and encourage further quantitative research into the role of fidgeting in ADHD.

## 1 Introduction

Attention-Deficit/Hyperactivity Disorder (ADHD) is a neurodevelopmental disorder characterized by clinically impairing levels of inattention, hyperactivity and impulsivity (1). ADHD often leads to chronic functional impairments in various areas such as family, school, and social contexts (1). Adults with ADHD are particularly prone to higher rates of anxiety, depression, and increased daytime sleepiness (2–6). The prevalence of ADHD varies by region and time period. However, there has been an increase in the worldwide diagnosis estimate. In 2007, a metastudy reported an estimate of 5.3% (7) while in 2023, another metastudy reported an estimate of 8.0% of children and adolescents diagnosed with ADHD (8). Many of these cases continue into adulthood. A 2016 study estimated that 60% of children with ADHD continue to be significantly impaired by symptoms as an adult (9, 10).

Hyperactivity, notably, is regarded as a cardinal feature of the disorder and constitutes the bulk of symptoms identified within the hyperactivity–impulsivity domain of DSM–5 ADHD criteria (1). Hyperactivity in young children often manifests as excessive running, climbing, and leaving their seat. In adults with ADHD, however, hyperactivity tends to be more subtle and is characterized by fidgeting (11). Fidgeting is defined as non–goal–directed actions undertaken for stimulation (12). It is characterized by repetitive or patterned movements that can be categorized into two types: extrinsic and intrinsic. Extrinsic fidgeting involves interaction with external objects, such as a pen, while intrinsic fidgeting, which is the focus of this study, involves movements made with one’s own body, such as hand, arm, and leg movements.

As referenced in (13), early theories suggested that fidgeting, and hyperactivity in general, is a ubiquitous trait unaffected by environmental or other factors (14, 15). Empirical findings using objective measurement devices have shown that the average activity level is higher for individuals with ADHD compared to controls (16, 17). However, other theories proposed that hyperactivity, specifically fidgeting, varies under different conditions, environments, and workloads. These theories stated that fidgeting serves as an involuntary mechanism for self–regulating attention and enhancing alertness, especially during tasks perceived as cognitively demanding or monotonous (18–20). Authors have hypothesized that excessive fidgeting may be indicative of poorly regulated noradrenergic and dopaminergic systems, along with altered activity in the motor areas of the brain (21, 22). Fidgeting may serve as a mechanism to increase stimulation during cognitively demanding tasks. For instance, children with ADHD fidget more compared to typically developing children under cognitively demanding conditions (23–25). A related study (26) observed an increase in fidget intensity during correct trials on the Flanker task for children with ADHD, while no significant difference between trials was found for typically developing children. Another study (27) observed increased movement during the Flanker compared to a less cognitively demanding task. Flanker is a cognitive task that measures response inhibition in a setting of selective visual information and is well suited as a tool to control cognitive load (28).

Fidgeting behavior has also been shown to be related to reaction time variability (29, 30), a proposed core neuropsychological deficit in ADHD (see (31) for review). Many studies (29, 32–34) confirm that both children and adults with ADHD exhibit higher reaction time variability. However, the mean reaction time has shown conflicting results for children and adults. Some studies (32, 35, 36) indicate that individuals with ADHD have a higher mean reaction time (slower processing), compared to controls. Other studies (29, 30) have found insignificant differences. It has been noted that some extremely slow responses and extremely fast responses, which are more likely in individuals with attention impairment and impulsive tendencies, have skewed the mean for ADHD samples. As such, the true reaction time has been shown to be better modeled by an Ex–Gaussian distribution (29), which takes into account these outliers.

Many studies have continued to explore the role of fidgeting in ADHD through actigraphy (16, 17, 37, 38) and other measurement tools, such as eye movement (39), to quantify movement and fidgeting. However, there are currently no standard variables to define fidgeting. Various studies report different variables to quantify movement, often resorting to device–defined features (16, 17, 37, 38, 40–43), which reduces replicability when other devices are used. This is particularly concerning as significant discrepancies have been observed in data recorded by two different actigraphy devices when used on children (44). Additionally, several studies used a large number of variables for the application of machine learning techniques, with (45), (46), and (47) reporting the use of 117, 112, and 45 features, respectively. While this approach may enhance the goodness of fit to certain variables, it decreases interpretability of fidgeting. Conversely, some studies have focused solely on one variable (26, 48), which, while enhancing interpretability, may not comprehensively capture different aspects of fidgeting.

The primary objective of this study is to better understand fidgeting in ADHD and its relation to performance on a cognitive task, specifically the Flanker (49) task. Furthermore, while many existing studies focus on ADHD in children, our study specifically targets adults, a demographic that is less explored. We formulate several hypotheses to test through our experiments. In line with the theory that fidgeting may serve as a compensatory mechanism during cognitively demanding conditions, we expect to observe a change in movement patterns, especially as the task progresses into the later stages. We also expect fidgeting to be more pronounced in individuals with more severe ADHD symptoms. Furthermore, while most of our adult population may not struggle with accuracy on the Flanker task, we expect a larger range of reaction time and reaction time variability for participants with more severe ADHD ratings. These differences, in both fidgeting, reaction time, and reaction time variability could provide insights into fidgeting and its relationship with ADHD during cognitively demanding tasks.

## 2 Methods

### 2.1 Participants

This study included 70 adults (36% male), between the ages of 18 and 50 years (average 30.5 years), diagnosed with any presentation type of ADHD. Participants were recruited through a social media campaign (e.g., Facebook, Instagram) from the UC Davis Health Department of Public Affairs and Marketing, local clinics at UC Davis, the institutions’ electronic health system and the UC Davis MIND Institute’s research registry, and from previous studies. Participants were initially screened via a video conference call to confirm ADHD diagnosis using the Adult Mini International Neuropsychiatric Interview (MINI) (50) for DSM 5–TR (1). Participants were excluded if they met any of the following criteria: currently prescribed psychoactive medication, with the exception of stimulant medication for ADHD or medication that can affect heart rate, presence of significant depression or psychotic disorders based on the Adult MINI DSM 5 and confirmed by study team clinician or clinician trainees, presence of autism diagnosis, or visual or hearing impairment or any other disorder that may interfere with task performance. A licensed psychologist with extensive experience diagnosing ADHD evaluated initial phone screening data to determine initial eligibility and final determination for study inclusion or exclusion. Participants who met DSM 5–TR ADHD criteria and endorsed “fidgeting with hands or feet or squirm in seat” as occurring “often” or “very often” were invited to proceed to the in–person assessment. The Wechsler Abbreviated Scale of Intelligence (WASI) Digit Span subtest was also given at this point to be used as a blocking variable for randomization purposes to the two study groups in regard to working memory performance related to another aspect of the study not discussed in this article. Inclusion criteria included 1) IQ ≥ 80, 2) aged 18–50 years, 3) met DSM–5–TR ADHD criteria based on the MINI DSM 5 interview, 4) with current (self and observer) and retrospective (caregiver) ratings on the Barkley Adult ADHD Rating Scale (BAARS) (51) to provide additional information to determine if they met study criteria. Participants prescribed stimulant medication were asked to participate in the in–person assessment at times when they were on medication breaks and not taking the medication that day, typically at least 24 hours before the testing session began. Informed written consent was obtained from all participants. The University of California, Davis Institutional Review Board approved the study.

The present study was part of a larger study that investigated the effect of using a fidgeting ball in ADHD on a variety of cognitive and emotional tasks, thus participants were randomized to a condition with a computerized fidgeting device or no fidgeting device (2:1) based on their working memory performance (Digit Span backward from WAIS–IV) and sex assigned at birth.

### 2.2 Data collection

#### 2.2.1 Measures

##### 2.2.1.1 Flanker task

**Figure 1:**
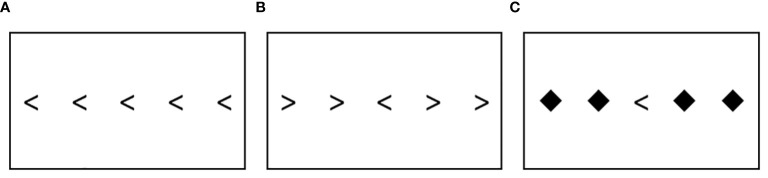
The Flanker task: **(A)** Congruent trial **(B)** Incongruent trial **(C)** Neutral trial.

The main task used in this study was the Flanker task (28), which has a lengthy history of use in ADHD studies (e.g., 26, 31, 50, 52–55). The Flanker task is a response inhibition and attention task, which requires participants to select the correct orientation of the central arrow (i.e., the target) from a list of five arrows, with 4 of the arrows on the two sides considered flankers. In each trial, stimuli are displayed for 1,000 milliseconds preceded by a 1,000 millisecond inter–trial interval (ITI) with a black fixation cross. The task includes three trial types – congruent, incongruent, and neutral as shown in **Figure 1**. In congruent trials, the target arrow and flanker arrows share the same orientation (**Figure 1A**), while in incongruent trials flanker arrows point to the opposite direction of the target arrow (**Figure 1B**). Neutral trials involve flanker arrows taking neutral shapes (**Figure 1C**). There are 40 trials in each trial type, with 120 trials in total. Participants were given a button box that had two buttons to be used in the task with their right hand. The first (left) button was to be pressed with their index finger when the target arrow was pointing to the left. The second (right) button was to be pressed with their middle finger when the target arrow was pointing to the right.

In our analysis, we use the total number of correct trials (out of 120), reaction time, and reaction time variability. When calculating reaction time and time variability, a standard Gaussian distribution may not adequately represent participants exhibiting ADHD symptoms. Impulsive symptoms may cause extremely fast responses, while inattentive symptoms may lead to extremely slow responses. Consequently, first and second order statistics could be skewed and may not accurately capture outliers. The exponentially modified Gaussian (ex–Gaussian) distribution is more appropriate for representing reaction time and its variability as shown in previous studies (29, 56–58).

The ex–Gaussian distribution comprises two components: an exponential curve and a Gaussian curve. There are three parameters that describe these components: ‘mu’ is the mean of the Gaussian, ‘sigma’ is the standard deviation of the Gaussian, and ‘tau’ is the mean and standard deviation of the exponential. Therefore, ‘mu’ represents the average reaction time, while ‘sigma’ and ‘tau’ represent the variability of the reaction time. We adopt an implementation from prior research (56) to fit the reaction time into an ex–Gaussian distribution.

##### 2.2.1.2 Stroop task

The Stroop Color and Word Test (58) was also administered. The task tests response inhibition. Previous studies (59–61) have shown performance on the Stroop is compromised in the ADHD population. In the task, participants were required to read two tables as fast as possible. The first table presents color patches, and the second “color–word” table presents mismatched words of color and printed ink. Participants were given 45 seconds to name the colors on each table. The number of items they successfully completed on each was recorded. Actigraph data from this task was not utilized in this study.

##### 2.2.1.3 Barkley adult ADHD rating scale–fourth edition

ADHD symptoms were measured using the BAARS–IV (51). The BAARS–IV: Other–Report: Childhood Symptoms requires an observer (i.e., caregiver) to rate behaviors from when the participant was between ages 5 and 12 and the BAARS–IV: Self–Report: Current Symptoms requires participants to rate their behaviors from the past 6 months. The BAARS–IV has satisfactory internal consistency and test–retest reliability over a 2– to 3–week period (51). Both versions consist of 18 items on a 4–point scale, ranging from 1 (never or rarely) to 4 (very often). The total score is the sum of all 18 items, resulting in a possible score of 18–72.

##### 2.2.1.4 Barkley deficits in executive functioning scale

The BDEFS (62) is an 89–item self–report measure of executive functioning deficits in everyday life. The BDEFS has high internal consistency and test–retest reliability (63). Each item is answered on a 4–point scale (1 not at all, 2 sometimes, 3 often, 4 very often). Only the self–report from items 77–89 from the Self–Regulation of Emotion domain (Section 5) were administered. The total score is the sum of these 13 items, resulting in a possible score of 13–52.

##### 2.2.1.5 Affective reactivity index

The ARI (64) is a self–rated measure of irritability that is validated for youths ages 6–17 years and has also been used with adults (65–69). It has excellent internal consistencies in several clinical and nonclinical groups (α’s>0.88). It consists of six items rated on a 3–point scale (“not true,” “somewhat true,” and “certainly true,” scored 0, 1, and 2, respectively). The total score is the sum of these six items, resulting in a possible score of 0–12.

#### 2.2.2 Actigraph and Other Devices

Intrinsic fidgeting was objectively measured using Actigraph devices. Two transducers made by Mindware Technologies (Gahanna, OH) were attached to the participants’ left wrist and the right ankle. The actigraphy device was attached to the left wrist ensuring it was available even during tasks that required responses to be registered with the right hand. Participants were required to use their right hand to press a button box to respond to stimuli during the Flanker task. Therefore, the actigraphy device was attached to the left wrist to ensure that movements during the Flanker task were not directly confounded by the button presses from the right hand. The right hand was used to hold the button box as most participants were right–hand dominant. We were not able to individualize the placement of the button box (i.e., move it for the left–hand dominant participants) because of restrictions imposed by the physical set–up of the equipment necessitating that the button box be placed in fixed and close proximity to other testing equipment. Another actigraphy device was attached to the right ankle to cover more laterality of movement. Given that only two devices were used, and one was attached to the left wrist, this set–up aimed to record movement from both sides of the body, while removing the impact of the button press from the right hand on the actigraph–recorded movements.

Data were sampled at 500 Hz for 3 coordinates X, Y, and Z to capture movement and acceleration from 3 dimensions. Actigraph data were synchronized with the tasks’ events to allow detailed trial–by–trial analysis. While various tasks targeting working memory, processing speed, response inhibition, emotion induction, and stress were administered, this study utilized data from only the Flanker task.

The data collected for this study was based on an ongoing larger project that explores intrinsic fidgeting with the arms and legs as well as extrinsic fidgeting with a fidget ball. While actigraphy was collected from all participants, there was a ratio of 1:2 between no–fidget ball and fidget ball groups, in which participants in the fidget ball group were given a fidget ball with electronic sensors to hold.

### 2.3 Fidget Variables

As we are focused on fidgeting as a repetitive and patterned movement, it is important to accurately distinguish it from other movements. In the current context of this study, we have collected data in a controlled setting in which participants are stationary in their chairs. Furthermore, with the exception of the Flanker task, the included experimental tasks do not require repetitive movement. While the Flanker does require a press on the response button in their hand, only a single press is required during each trial. We consider each Flanker trial as an independent epoch, meaning that a single press within a trial is not considered repetitive movement in the data. We thus focus on repetitive motion as a characteristic marker of fidgeting, regardless of direction.

**Figure 2:**
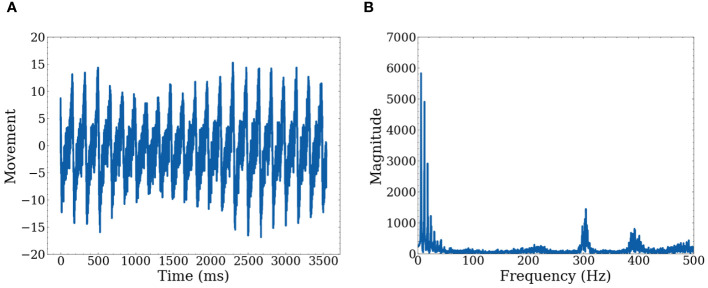
Graphs of raw data for one axis **(A)** Shows movement with respect to time on a single axis **(B)** Shows movement represented in the frequency domain after FFT.

However, it is difficult to determine repetition given the raw actigraph data, as this provides only location at specific timeframes. We transformed the raw data to movement data by taking the derivative with respect to time for each axis (X, Y, Z) separately. A graph of a single axis is shown in **Figure 2** of both the time domain and the frequency domain. Note that we record actigraphy data at a high frequency of 500 Hz. While there is valuable information in the high frequencies, when focusing on extracting repetitive motion, these high frequencies can be treated as unwanted artifacts.

**Figure 3:**
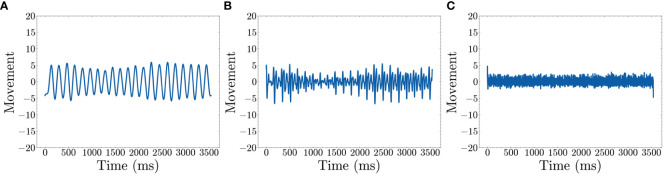
Movement after filters (time domain) **(A)** Shows the recreated movement graph using only *1~20 Hz* **(B)** Shows the recreated movement graph using *20~100 Hz* **(C)** Shows the recreated movement graph using *100~500 Hz.*.

We collected calibration data before the analysis to confirm that low frequencies accurately represent repetitive movement. We conducted three types of movement measurements, each lasting a few minutes to ensure consistency and accuracy. The first calibration involved no movement to confirm that no movement is detected when the participant is stationary. The second involved simulated fidgeting, such as leg movement and table tapping with the hands. The final calibration involved rapid movement to confirm that such movements are detected, but the signals are more erratic and stronger than fidgeting. **Figure 3** shows data from simulated fidgeting. In addition, there is a limit to how fast people can move, thus for repetitive movement, high frequencies may not hold important information. The use of low frequencies is consistent with previous studies showing that significant differences between ADHD subtypes (70) and increased movement during working memory tasks in children with ADHD (13) are found in the low frequencies.

The Fast Fourier Transform (FFT) is applied to decompose the movement data into the frequency domain which represents data as repetitive sine waves of differing frequencies (71). This enables the identification of dominant frequencies associated with repetitive movements. To visually represent this, we used simulated fidgeting calibration data to show a frequency domain graph of dominant frequencies. Peaks in **Figure 3B** show dominant frequencies for defining fidgets. Based on the figure, we select 1~20 Hz as the representative data for defining fidgeting as these are the most dominant frequencies. The frequencies are converted back to the time domain using an inverse FFT algorithm (71). As the selection of frequencies may change with other datasets, we leave this as a hyperparameter.

The resulting signals within the 1~20 Hz range, shown in **Figure 3A**, represent low–frequency sinusoidal waves. These waves are characterized by their repetitive and continuous nature, which aligns with our definition of fidgeting as non–goal–directed repetitive movements. Note that **Figure 3B** shows peaks around 300 and 400 Hz. However, **Figure 3C** reveals that the signal in this range is mostly noise, possibly from the actigraphy device itself. Additionally, **Figure 3B** illustrates movement information within the 20~100 Hz range. As our goal is to quantify general fidgeting, the smooth waves from the lower frequencies are preferred over the less smooth waves in the higher frequency range.

**Figure 4:**
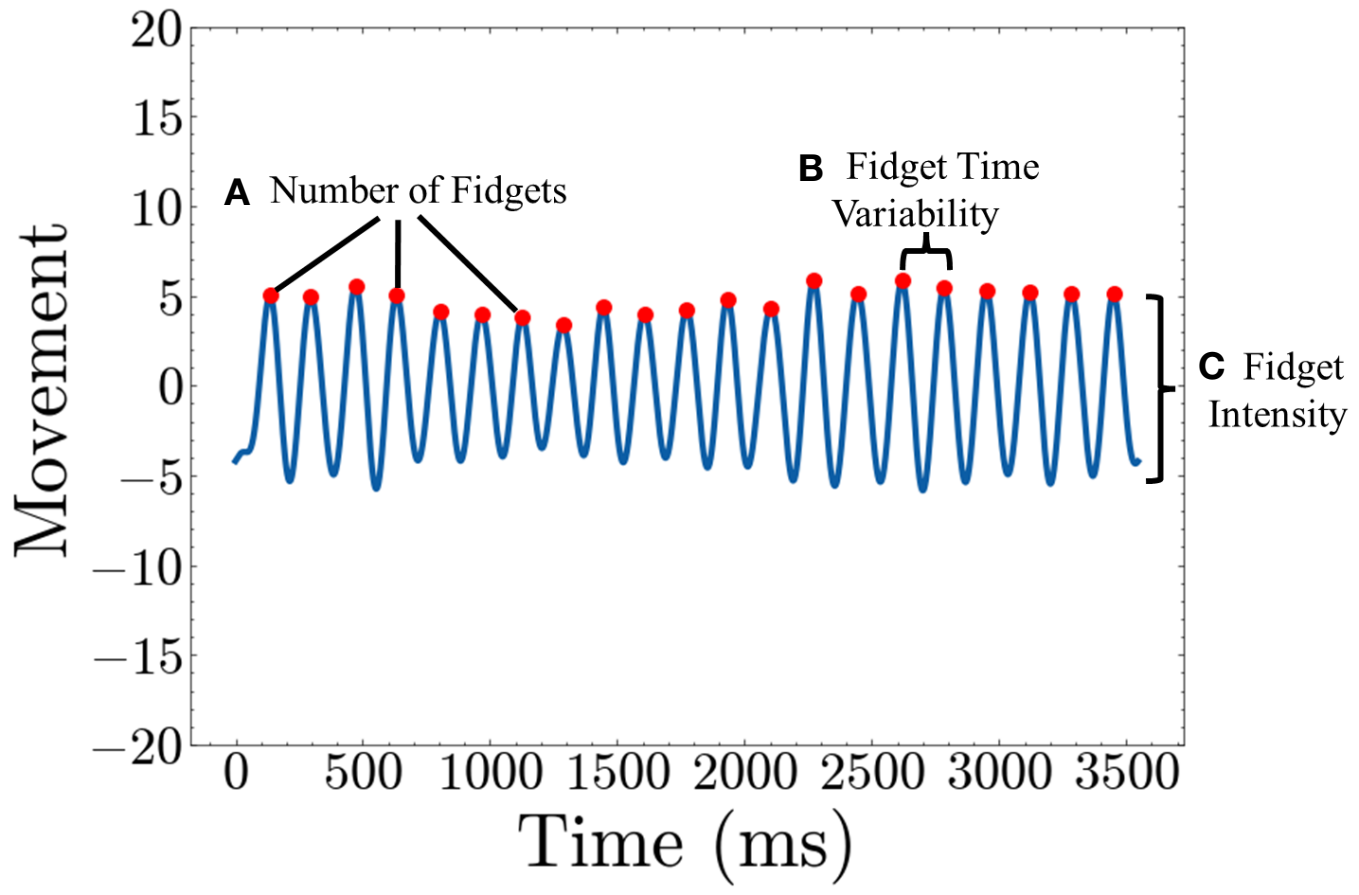
Quantifying Fidget variables **(A)** *Number of Fidgets* is calculated by counting the number of peaks in the movement graph **(B)** *Fidget Time Variability* is calculated by taking the average of variance of the time between consecutive peaks **(C)** *Fidget Intensity* is calculated by taking the average height of each peak.

With these data from 1~20 Hz, we have established three straightforward and representative fidget variables based on peaks and valleys in the low–frequency movement data. The “Number of Fidgets” refers to the count of peaks within a specified time frame, indicating the frequency of repetitive movements. “Fidget Variability” describes the variance in time intervals between peaks, with a higher value suggesting intermittent fidgeting behavior characterized by pauses in fidgeting. Lastly, “Fidget Intensity” is defined as the average height of the peaks in the movement data, with a higher value suggesting more intense movements during fidgeting. A visual representation is shown in **Figure 4**. As we use the actigraphy data synchronized with the Flanker task, we generate all three variables for each of the 120 trials and subsequently average these values. This process is similar to the many studies that epoch actigraphy data (16, 17, 37–39, 41–43).

We have designed this framework to quantify meaningful intrinsic fidgeting variables for a comprehensive comparison in the Flanker task. Our fidgeting analysis algorithms and code are made publicly accessible to ensure reproducibility and assist other researchers to analyze fidgeting more systematically. By making our code publicly available and analyzing data from adults, we hope to encourage future studies to further our understanding of fidgeting and its relevance to ADHD across different age groups.

## 3 Results

### 3.1 Demographics and characteristics

**Table 1** presents the demographics, characteristics, and rating scale scores of the study participants. This study included 70 individuals, all diagnosed with ADHD. Non–ADHD participants were not included in the study as the goal was to assess the relation between fidgeting and ADHD symptoms. Additionally, 70% of the participants were provided with a smart fidget ball to record touches, but data from the fidget ball were not used in this study. As verified in the **Supplementary Materials (Table A)**, the possession of the fidget ball did not significantly influence population averages, with the exception of gender and the *Fidget Time Variability* fidget variable. We focus primarily on the variables that remained unaffected by the use of the fidget ball. These results focused only on data from actigraphy devices attached to the wrist and ankle.

**Table 1:** Demographic and self–reported rating scales of adults with ADHD (n=70).

Characteristics	ADHD (n=70)
Gender (Male Ratio)	36% Male
Age	30.5 (7.2) years
Has Fidget Ball	70%
BAARS (Self Report)	51.6 (9.3)
BDEFS (Self Report) [Section 5: 77~89]	27.8 (7.2)
ARI (Self Report)	8.8 (2.5)

*BAARS, Barkley Adult ADHD Rating Scale; BDEFS, Barkley Deficits in Executive Functioning Scale; ARI, Affective Reactivity Index.

Furthermore, various scales, including the BAARS, BDEFS, and ARI were used to assess the relation between fidgeting and symptoms related to ADHD in the real world. The sum of scores from each scale was used as a comparison metric to the severity of ADHD symptoms. **Table 2** shows statistics related to the performance and reaction time and **Table 3** shows data from actigraphy data during the Flanker task.

**Table 2:** Performance and reaction time statistics on the flanker task.

Flanker	Mean (STD)	Percentage
Total Score (120 trials)	115.5 (7.5)	96.3%
Incorrect (120 trials)	3.6 (6.4)	2.96%
Omission Errors (120 trials)	2.7 (14.7)	2.21%
Congruent Correct (40 trials)	39.7 (0.7)	99.1%
Incongruent Correct (40 trials)	36.3 (7.0)	90.8%
Neutral Correct (40 trials)	39.5 (1.2)	98.7%
Reaction Time (Mu)	369.3 (46.2)	–
Reaction Time Variability (Sigma)	51.9 (18.4)	–
Reaction Time Variability (Tau)	70.0 (26.5)	–

**Table 3:** Quantified fidgeting variable statistics in adults with ADHD.

Fidget Variables		ADHD (n=70)
Arm	Number of fidgets per trial	2.1 (1.5)
	Fidget time variability	131.3 (103.7)
	Fidget intensity	0.1 (0.2)
Leg	Number of fidgets per trial	2.6 (1.4)
	Fidget time variability	135.5 (80.4)
	Fidget intensity	0.5 (0.9)
Arm + Leg	Number of fidgets per trial	4.7 (2.3)
	Fidget time variability	266.9 (128.4)
	Fidget intensity	0.6 (1.0)

### 3.2 Fidget variables – Flanker

#### 3.2.1 Performance

**Figure 5:**
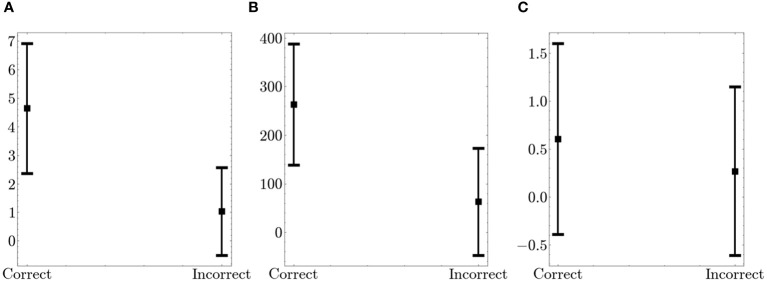
Comparison of Fidget Variables between Correct and Incorrect Trials on the Flanker. The figure displays the average values (squares) and the standard deviation (bars in both directions) **(A)** *Number of fidgets per trial* **(B)** *Fidget variability* **(C)** *Fidget intensity*.

We conducted a comparison of fidget variables in relation to performance on the Flanker task. **Figure 5** presents the combined fidget variables from the arm and leg for both correct and incorrect trials. Note, only 56 participants were considered in this comparison as 14 participants did not have any incorrect trials. There were significant differences for all variables. T–scores were 10.72 (p<0.001), 9.84 (p<0.001), and 2.08 (p=0.04) for Number of fidgets per trial, Fidget variability, and Fidget intensity, respectively. The high t–scores show that there was significantly more fidgeting, with more variability and intensity during correct trials when compared to incorrect trials. However, the small number of incorrect trials may cause non–conclusive interpretation.

**Figure 6:**
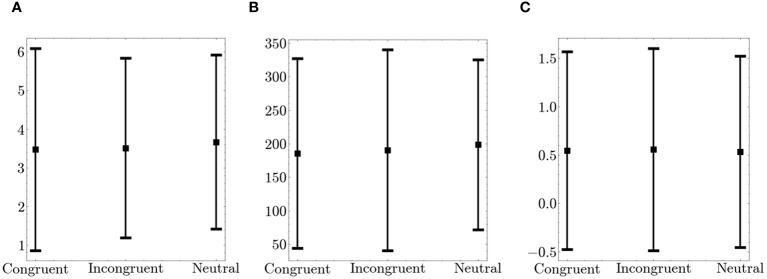
Comparison of Fidget Variables between Congruent, Incongruent and Neutral Trials on the Flanker. The figure displays the average values (squares) and the standard deviation (bars in both directions) **(A)** *Number of fidgets per trial* **(B)** *Fidget variability* **(C)** *Fidget intensity*.

Furthermore, we examined fidget variables across congruent, incongruent, and neutral tasks. **Figure 6** shows the summed fidget variables from the arm and leg. There were no significant differences in fidgeting behavior across the different trial types.

#### 3.2.2 Time effect

We conducted a comparison of fidget variables over time, specifically comparing the fidget variables from the first 30 trials (Q1) and the last 30 trials (Q4), as shown in **Table 4**. There were significant differences in the *Number of Fidgets* and *Fidget Variability* variables. The high positive t–scores for these variables show that there was significantly more fidgeting that was more variable during the last 30 trials when compared to the first 30 trials. Performance on the Flanker, as shown by the number of correct trials, did not show significant differences.

**Table 4:** Comparison of fidget variables and flanker performance between the first (Q1) and last (Q4) 30 trials.

Fidget Variables		Q1 (First 30 trials)	Q4 (Last 30 trials)	t–value
Arm+ Leg	Number of fidgets per trial	2.3 (2.0)	3.5 (2.5)	2.89**
	Fidget time variability	130.5 (120.8)	187.1 (144.7)	2.46*
	Fidget intensity	0.4 (1.0)	0.5 (1.0)	0.28
Flanker				
	Correct trials on Flanker	28.7 (2.2)	28.3 (4.1)	0.58

*p<0.05, **p<0.01, Mean (STD) are shown.

### 3.3 Group differences for time effect

#### 3.3.1 Reaction time

We explored the time effect from the Flanker task further by exploring group differences. Participants were divided into equal groups based on the median of the population set as the threshold, as shown in **Table 5**. There were significant differences for certain variables within the High Mu (high mean reaction time), and Low Sigma and Tau (low reaction time variability) groups.

**Table 5:** Comparison of fidget variables and flanker reaction time and reaction time variability between the first (Q1) and last (Q4) 30 trials.

Fidget Variables		Mu (Average reaction time)					
		Low (n=35)			High (n=35)		
		Q1	Q4	t	Q1	Q4	t
Arm+Leg	Number of fidgets per trial	2.7 (2.2)	3.6 (2.6)	1.53	2.0 (1.8)	3.4 (2.4)	2.62*
	Fidget time variability	148.9 (130.9)	173.0 (138.6)	0.72	112.1 (106.6)	201.3 (149.2)	2.79**
	Fidget intensity	0.6 (1.3)	0.6 (1.2)	0.18	0.3 (0.6)	0.4 (0.6)	0.28

		Sigma (Variability of reaction time)					
		Low (n=35)			High (n=35)		
		Q1	Q4	t	Q1	Q4	t
Arm+Leg	Number of fidgets per trial	2.1 (3.5)	3.5 (2.5)	2.51**	2.5 (2.2)	3.5 (2.5)	1.58
	Fidget time variability	120.4 (188.8)	188.8 (138.5)	2.20*	140.7 (127.2)	185.4 (150.6)	1.30
	Fidget intensity	0.3 (0.4)	0.4 (0.7)	0.77	0.6 (1.2)	0.6 (1.2)	0.15

		Tau (Variability of reaction time)					
		Low (n=35)			High (n=35)		
		Q1	Q4	t	Q1	Q4	t
Arm+Leg	Number of fidgets per trial	2.4 (2.0)	3.6 (2.2)	2.37*	2.3 (2.0)	3.3 (2.7)	1.74
	Fidget time variability	136.2 (124.8)	182.8 (120.4)	1.54	124.9 (116.3)	191.5 (165.3)	1.89
	Fidget intensity	0.5 (1.2)	0.6 (1.2)	0.54	0.4 (0.9)	0.4 (0.7)	–0.3

*p<0.05, **p<0.01,

Q1 = first 30 trials, Q4 = last 30 trials,

Mean (STD) are shown.

In order to gain more understanding of the groups, we analyzed the relation between fidgeting and other variables associated with ADHD. The task variables represent the overall scores from those tasks. Rating scale scores represent cumulative scores from all questions. As shown in **Table 6**, there was an association between the groups and performance of tasks. Mu groups demonstrated significant differences in performance on the Digit Span, Stroop color, and Stroop color–word performance. Sigma groups showed significant differences in the Stroop color–word performance. Tau groups showed significant differences in Stroop color–word. However, these groups were not correlated with rating scale scores.

**Table 6:** Comparison of Gender, Task Performance, and Self–reported Rating Scales between Low and High Reaction time and Reaction Time Variability.

		Mu			Sigma			Tau		
	Variables	Low	High	t	Low	High	t	Low	High	t
	Gender	0.6 (0.5)	0.6 (0.5)	0.68	0.6 (4.8)	0.6 (0.5)	0.14	0.5 (0.5)	0.7 (0.5)	–1.8
T a s k s	Digit span	11.7 (3.1)	10.1 (3.1)	2.15*	11.5 (3.6)	10.3 (2.6)	1.59	11.6 (3.1)	10.2 (3.1)	1.9
	Stroop Color	74.0 (13.9)	66.7 (10.5)	2.39*	72.7 (14.3)	68.0 (10.8)	1.52	70.2 (14.1)	70.6 (11.5)	–0.12
	Stroop Color–Word	52.1 (9.1)	47.4 (8.3)	2.20*	52.0 (8.8)	47.4 (8.6)	2.17*	52.1 (9.8)	47.3 (7.3)	2.29*
S c a l e s	BAARS	51.2 (10.7)	52.1 (7.9)	–0.39	50.7 (8.5)	52.6 (10.2)	–0.86	50.6 (10.1)	52.8 (8.6)	–1.0
	BDEFS	27.9 (8.0)	27.6 (6.0)	0.2	27.5 (6.9)	28.0 (7.2)	–0.30	27.6 (7.4)	27.9 (6.7)	–0.2
	ARI	8.8 (2.6)	8.8 (2.4)	–0.05	8.7 (2.3)	9.0 (2.6)	–0.53	8.6 (2.0)	9.0 (2.9)	–0.73

*p<0.05, Mean (STD) are shown.

### 3.4 Correlation analysis to scale scores

We applied a linear model to fit each of the scales. The goal of using a linear model is to assess the correlation between a set of variables – the fidget variables, and a target variable – the rating scale scores. Additionally, a linear model allows us to measure correlation not only between a single fidget variable and rating scales, but the combination of the fidget variables. The fidget variables are designed to capture different factors of fidgeting, thus considering the combination allows a more comprehensive measure. Furthermore, in any goodness–of–fit measure, it is important to have a baseline measure. We also assess the fit between task performance and rating scale scores to establish a baseline.

To ensure a fair comparison, we used 6 variables for each linear model. Task performance measures included, Digit Span (working memory tasks), Stroop color and color word, Flanker, and reaction time (Mu) and reaction time variability on the Flanker (Tau). In the case of Fidget Variables, we included variables from the arm and leg separately. Note that fidget variables are extracted from actigraph data during only the Flanker task, while Task Performance involves variables across various tasks. As shown in **Table 7**, the fidget variables showed a slightly better fit to the rating scale scores.

**Table 7:** Correlation of self–reported rating scales to task performance and fidget variables.

Scale	Task Performance R (p)	Fidget Variables R (p)
BAARS (Self Report) [1~30]	0.34**	0.52**
BDEFS (Self Report) [Section 5: 77~89]	0.32*	0.39**
ARI (Self Report)	0.30*	0.37**

*p<0.05, **p<0.01.

## 4 Discussion

The primary objective of this study is to better understand fidgeting in adults with ADHD and its relation to performance response inhibition, attention and overall cognitive control, using a Flanker task. This was achieved by creating new variables intended to represent fidgeting. Our results, based on the newly defined fidget variables, support the theory that hyperactivity is not ubiquitous but rather that hyperactivity, particularly fidgeting, may serve an unconscious function and emerge as a response to particular environments. Our results demonstrate a significant increase in all fidgeting variables during correct trials. Participants exhibited more fidgeting during correct trials compared to incorrect trials. Specifically, fidgeting occurs more frequently, with less consistency, and more intensely during successful task performance. For adults, it is possible that fidgeting serves as an unconscious method to boost arousal and alertness to enable them to focus more on the task stimuli and keep pace with it, thus performing better when fidgeting. It may also allow them to be less distracted by extraneous stimuli and heighten focus on the critical stimuli of the task, such as the central arrow in the Flanker task.

These results align with previous studies that also observed increased movement during the Flanker task. One study (26) reported similar findings, demonstrating more intense movement during correct trials in children. Another study (27) showed increased movement during the Flanker compared to a less cognitively demanding task. We hypothesize that fidgeting may also enhance arousal when a person with ADHD is under–stimulated or even bored and the person is trying to maintain attention and engagement (20, 21, 72). This hypothesis is supported by the comparison of movement between Q1 (first 30) and Q4 (last 30) trials. We observed significant differences in fidgeting when comparing Q1 and Q4 trials, with the *Number of Fidgets* variable showing higher values in Q4. The increased fidgeting during the late trials may indicate difficulty sustaining attention, particularly as a task becomes more routine, and fidgeting could be aiding in arousal. This hypothesis is corroborated as we see no significant differences between performance in early and late trials, as shown in **Table 4**. An early study by our team assessing rates of actigraph movement in young boys with ADHD versus typically developing boys (73) are consistent with findings from this study in that rates of movement increased over time. In this Schweitzer & Sulzer–Azaroff (73) study, we also found that giving access to external stimulation via toys and music reduced actigraph movements, suggesting that the intrinsic fidgeting movements serve to increase stimulation, when needed.

In contrast to a previous study (74), we did not compare consecutive trials but compared groups of trials similar to (26). This involved a comparison of correct and incorrect trials, and Q1 and Q4 trials. It is important to note that most adults do not struggle in regard to accuracy on the Flanker task, with an average of only 4.5 errors out of 120 trials. While there was a significant increase in all fidgeting variables during correct trials, which confirms findings from a previous study (26), the high accuracy rates may suggest that the fidget variables for incorrect trials may not be the best estimate of how participants fidget during incorrect trials. We thus explored the relation between reaction time and fidgeting in Q1 and Q4 to gain further insights. Considering that reaction time and reaction time variability are major factors associated with ADHD in both children and adults and represent attentional readiness, we divided the groups based on the median Mu (reaction time), Sigma (reaction time variability), and Tau (reaction time variability). The *Number of Fidgets* variable showed a significant increase in Q4 compared to Q1 for one group within each reaction time variable. Specifically, participants with high reaction time (high Mu) and low reaction time variability (low Sigma and low Tau) fidgeted more during the later trials.

A meta–analysis on reaction time variability in ADHD (75) found individuals with the disorder generally exhibit higher reaction time variability compared to controls, while average reaction time is inconsistent across studies. Focusing on variability of reaction time, we found an association between higher variability with greater symptom severity within our participants with ADHD. Conversely, a lower reaction time variability suggested better sustained attention. We find that the low reaction time variability group, those able to better sustain attention, demonstrated significantly more fidgeting in Q4. This pattern further supports the theory that fidgeting aids in arousal, helping sustained attention.

However, because our study design limits any causal conclusions, it is important to acknowledge other possible interpretations. One possibility is that fidgeting is a by–product, rather than a contributor, to improved attention. In other words, it may be possible that the increase in cognitive effort needed to pay attention leads to an increase in fidgeting. While this interpretation is possible, there is overwhelming evidence from experimental studies that show that cognitive functioning is superior during physical activity compared to conditions without physical activity (76–81). Prior research also indicates physical activity causes the release of neurotransmitters such as dopamine and norepinephrine (82) which, in turn, can improve task performance. The release of these neurotransmitters provides an explanation for the efficient attention observed through task performance, and the release of these neurotransmitters could be attributed to the increased fidgeting.

Lastly, a correlation analysis was performed using ADHD–related symptom rating scales. For each rating scale, linear regression models were fitted using fidget variables and task performances. The aim of this analysis was to validate the relevance of the fidget variables in relation to symptom severity as indicated by the rating scales. Baselines are important for interpreting correlations with subjective measures such as rating scales. Additionally, the number of variables were kept consistent throughout comparisons as additional variables allow for more variance of the target variable to be explained. In general, the fidget variables showed a stronger correlation with the rating scale scores compared to task performance variables. The correlation is moderate. We note that the fidget variables are quantified solely from the Flanker task, whereas task performance includes performance on various tasks. The task performance on the Flanker task alone has even weaker correlation. This highlights the effectiveness of the fidget variables in capturing meaningful associations with ADHD.

Defining these fidget variables is important, as there is currently no standard with many studies (16, 17, 37, 38, 40–43) using only variables automatically provided by actigraphy devices. With the increase in digital technologies, it is important to find standard variables that can be reproduced across devices and platforms. For instance, QBTech (83) attached an accelerometer to the head during a continuous performance task (CPT). Furthermore, advancements in virtual reality (VR) have contributed to ecological validity by simulating more realistic settings. One study (84) developed a virtual CPT in classroom settings in VR, while other recent studies have expanded the application of VR to simulate diverse settings presenting tasks that attempt to resemble everyday activities (85, 86). These digital devices are able to collect accelerometer movement data. These data–rich settings further emphasize the importance of understanding, defining, and quantifying fidgeting, independent of device–specific variables.

### 4.1 Limitations

Our study had several limitations. Firstly, the recruitment process resulted in a gender imbalance, with more female participants, particularly in the group that was given a fidget ball. Additionally, the *Fidget time variability* variable was lower in the fidget ball group. It would be ideal to separate these groups, with a sufficient number of participants. Secondly, impulsive movements may not be effectively captured by the defined fidget variables. However, as we have focused on fidgeting, we were specifically interested in the repetitive aspect of movement. Lastly, while the study’s findings are promising, it is important for future research to continue to conduct extensive testing and comparisons across various tasks. Specifically, given the high number of comparisons conducted in this study, there is a possibility that some variables might appear arbitrarily significant, a phenomenon that could occur in any study. Further exploration of fidgeting quantification is necessary especially because of the absence of a standard fidget quantification metric as a baseline for comparing.

## 5 Conclusion

In conclusion, our study suggests that fidgeting may serve a functional role in adults with ADHD, particularly in aiding sustained attention during attention–demanding, cognitive control processes. Our results reveal a significant increase in fidgeting during correct trials compared to incorrect trials on the Flanker task. Furthermore, participants with lower reaction time variability showed significantly more fidgeting during later trials. The presented data may indicate a potential compensatory role of fidgeting in adults with ADHD. Fidgeting could serve as a compensatory mechanism that aids sustained attention, especially during the latter stages of a cognitive task. If this is the case, it would be counterproductive to discourage adults with ADHD from fidgeting during cognitively demanding tasks such as during work meetings or class lectures. Rather, it may be beneficial to provide accommodations that allow and even facilitate adults with ADHD to fidget in a non–disruptive, but effective manner. Lastly, we validated our newly defined fidget variables through a significant correlation with ADHD rating scales. By making our code publicly available, we hope to encourage further research into the understanding of fidgeting and its relevance to ADHD.

## Data availability statement

The original datasets will be forthcoming in the National Institute of Mental Health Data Archive (https://nda.nih.gov/: C3606), however, these are not yet available at the time of publication as the data are part of a larger, ongoing project. Requests for the data supporting the conclusions of this article, will be made available by the authors, without undue reservation with requests for access directed to jschweitzer@ucdavis.edu.

## Ethics statement

The studies involving humans were approved by University of California, Davis Institutional Review Board. The studies were conducted in accordance with the local legislation and institutional requirements. The participants provided their written informed consent to participate in this study.

## Author contributions

HS: Conceptualization, Investigation, Methodology, Software, Validation, Writing – original draft, Writing – review & editing. CAC: Formal Analysis, Writing – original draft, Writing – review & editing. BF: Data curation, Project administration, Writing – review & editing. JFD: Data curation, Project administration, Writing – review & editing. SR: Formal Analysis, Methodology, Writing – review & editing. JB: Data curation, Project administration, Writing – review & editing. JBS: Conceptualization, Funding acquisition, Investigation, Supervision, Writing – review & editing. XL: Conceptualization, Formal Analysis, Supervision, Writing – review & editing.
