# Supplementary material for: A quantitative analysis of fidgeting in ADHD and its relation to performance and sustained attention on a cognitive task
Source: Front Psychiatry. 2024 Jul 1;15:1394096. doi: 10.3389/fpsyt.2024.1394096 (PMC11246969; doi:10.3389/fpsyt.2024.1394096)
Supplement: Supplementary file 1 [file Table_1.pdf]

Supplementary Material

1 Supplementary Table

Table A. Fidget ball membership differences

Characteristics		No Fidget Ball (n=21)	Fidget Ball (n=49)	t-value
Gender (Male Ratio)		40%	83%	4.24*
BAARS (Self Report) [1~30]		60.0 (9.3)	59.8 (9.0)	-0.08
BDEFS (Self Report) [Section 5: 77~89]		26.8 (6.0)	28.2 (7.4)	0.75
ARI (Self Report) [Affective Reactivity Index]		9.2 (2.5)	8.6 (2.4)	-0.84
**Flanker**				
Total Score		115.4 (8.1)	115.5 (7.2)	0.06
Incorrect		4.3 (8.1)	3.2 (5.4)	-0.66
Omission Errors		6.0 (25.5)	1.2 (4.1)	-1.24
Congruent Correct		39.7 (0.6)	39.7 (0.7)	-0.07
Incongruent Correct		36.3 (7.8)	36.3 (6.5)	0.02
Neutral Correct		39.4 (1.0)	39.5 (1.2)	0.27
Reaction Time (Mu)		355.6 (43.7)	375.0 (45.2)	1.63
Reaction Time Variability (Tau)		70.9 (25.0)	69.3 (26.7)	-0.23
**Task Performance**				
Digits		10.4 (2.9)	11.2 (3.3)	1.02
Stroop Color		69.4 (13.9)	70.6 (12.5)	0.35
Stroop Color-Word		50.4 (8.0)	49.6 (9.4)	0.36
**Fidget Variables**				
Arm+ Leg	Number of fidgets per trial	5.3 (2.2)	4.5 (2.3)	-1.42
	Fidget time variability	321.2 (115.4)	244.2 (125.4)	-2.32*
	Fidget intensity	0.7 (0.8)	0.6 (1.1)	-0.55

*p<0.05, **p<0.01
